# Study of the 16S microbiome of swans died during the H5N1 outbreak in the Caspian seashore

**DOI:** 10.3389/fvets.2025.1597890

**Published:** 2025-07-04

**Authors:** Kobey Karamendin, Sardor Nuralibekov, Temirlan Sabyrzhan, Yermukhammet Kasymbekov, Symbat Suleymenova, Aidyn Kydyrmanov

**Affiliations:** ^1^Laboratory of Biochemistry of Viruses, Department of Virology, Research and Production Center for Microbiology and Virology, Almaty, Kazakhstan; ^2^Laboratory of Viral Ecology, Department of Virology, Research and Production Center for Microbiology and Virology, Almaty, Kazakhstan

**Keywords:** swan, avian influenza, H5N1, post mortem, microbiome, Kazakhstan, Caspian Sea, mortality

## Abstract

**Introduction:**

In 2023 and 2024, mass mortalities of swans occurred on the Caspian coast of Kazakhstan, which affected more than seven hundred birds of a local population of 10–15 thousand. It is widely known that viral infections significantly affect the microbiome content of various organisms, but the influence of H5N1 infection in the gut microbiota of wild birds remains little studied. Almost no information is available on postmortem microbial changes after the devastating impact of H5N1 influenza.

**Methods:**

In addition to standard routine virological studies, we were interested in investigating the microbiological changes resulting from infection with the highly pathogenic H5N1 using 16S rRNA gene sequencing.

**Results:**

Virological studies of samples taken from the dead swans identified the highly pathogenic influenza virus H5N1 subtype as the primary cause of mortality. 16S analysis of samples from freshly dead swans revealed patterns of microbial dysbiosis caused by the overwhelming dominance of Campylobacter and Fusobacterium genera in the microbiome.

**Discussion:**

Unlike previous fecal microbiome studies in live H5N1-infected birds, this is the first post-mortem analysis revealing systemic dysbiosis across respiratory and digestive tracts in swans, dominated by Campylobacter (mean 74.7% ± 19.3) and Fusobacterium (mean 15.9% ± 12.2).

## Introduction

Influenza virus H5N1 is a highly pathogenic subtype that has a devastating effect on wild bird populations. This virus, which primarily infects the respiratory and gastrointestinal tracts of birds, has been identified in numerous wild bird species globally. Swans are particularly susceptible to H5N1, and infected birds often exhibit severe listlessness and neurological dysfunction consisting of seizures, tremors, and marked incoordination (1). Swans’ migratory behavior further exacerbates the virus’s dissemination, as they can carry and shed the virus over long distances, impacting other bird populations and ecosystems.

For the first time in Kazakhstan, a highly pathogenic avian influenza H5N1 outbreak significantly impacted wild swan populations in 2006 (2). This outbreak coincided with the seasonal migration of wild birds in the Caspian Sea, likely facilitating the virus’s spread across regions (3). Furthermore, mass mortality among swans caused by H5N1 was observed at Lake Karakol (Caspian seashore) in Kazakhstan during the winter of 2023 and 2024 (4). The Caspian Sea is not only an important crossing point for the migratory routes of wild birds, but also serves as a nesting and wintering area. Mass mortalities of gulls and terns regularly occur in this region due to H5N1 (5) and adenoviruses (6).

It is well established that infection with pathogenic viruses induces significant changes in the microbiome of the affected organism (7). Avian influenza virus (AIV) infection has been shown to reduce diversity and alter dominant taxa in the gut microbial composition in wild birds and poultry (8). In H5N1-infected swans, severe necrosis in multiple organs, along with lymphoid depletion in Peyer’s patches, which play a crucial role in the immune system, was observed (9).

In this study, we aimed to investigate the state of the swan microbiome following the lethal impact of highly pathogenic H5N1 in 2023 and 2024. The effect of H5N1 infection on the gut microbiota of migratory birds has been minimally studied (10); such research is crucial for understanding the microbiological processes that occur during epidemics.

## Materials and methods

### Sample collection

Swab samples were collected from swans (*Cygnus olor* and *Cygnus cygnus*) that died from confirmed H5N1 infection in December 2023 and December 2024 (Table 1). The samples were obtained from freshly deceased swans that died the same day or 1 to 2 days prior. The air temperature during sampling ranged from 0 to 2°C, and the carcass condition was assessed as very fresh. Two types of swab samples were obtained: cloacal and tracheal from juveniles and adults. The samples were collected using sterile swabs, placed in vials with transportation media, and stored for 1–2 days in liquid nitrogen at −195°C until delivery to the laboratory. In the laboratory, the samples were placed in a freezer and stored at −80°C until library preparation.

**Table 1:** Samples collected from dead swans in 2023 and 2024.

Sample	Swab Type	Age	Date	Cause
Swan-2023-1-juv-tr_S21_L001	Tracheal	Juvenile	27 Dec 2023	H5N1
Swan-2023-2-cl_S38_L001	Cloacal	Adult	27 Dec 2023	H5N1
Swan-2023-2-juv-tr_S20_L001	Tracheal	Juvenile	27 Dec 2023	H5N1
Swan-2023-3-cl_S39_L001	Cloacal	Adult	27 Dec 2023	H5N1
Swan-2023-3-tr_S18_L001	Tracheal	Adult	27 Dec 2023	H5N1
Swan-2023-6-cl_S41_L001	Cloacal	Adult	27 Dec 2023	H5N1
Swan-2023-6-tr_S19_L001	Tracheal	Adult	27 Dec 2023	H5N1
Swan-2023-10-cl_S42_L001	Cloacal	Adult	27 Dec 2023	H5N1
Swan-2023-11-tr_S22_L001	Tracheal	Adult	27 Dec 2023	H5N1
Swan-2024-2-juv-cl_S26_L001	Cloacal	Juvenile	07 Dec 2024	H5N1
Swan-2024-2-juv-tr_S29_L001	Tracheal	Juvenile	07 Dec 2024	H5N1
Swan-2024-2-tr_S25_L001	Tracheal	Adult	07 Dec 2024	H5N1
Swan-2024-3-cl_S23_L001	Cloacal	Adult	07 Dec 2024	H5N1
Swan-2024-4-cl_S30_L001	Cloacal	Adult	07 Dec 2024	H5N1
Swan-2024-5-cl_S24_L001	Cloacal	Adult	07 Dec 2024	H5N1
Swan-2024-5-juv-cl_S31_L001	Cloacal	Juvenile	07 Dec 2024	H5N1

### 16S rRNA gene amplification and sequencing

Microbial DNA was extracted using the PureLink Microbiome DNA Purification kit (Invitrogen, USA). The concentration of extracted DNA was assessed with a Qubit 4 fluorometer (Invitrogen, USA). The V3–V4 region of the 16S rRNA gene was amplified using the primer pair containing Illumina adaptors, following the PCR thermal cycling conditions recommended by the manufacturer (Illumina, USA). Amplicons were verified via agarose gel electrophoresis and purified with AxyPrep Mag PCR Clean-Up beads (Axygen, USA). Purified amplicons were indexed using the Illumina Nextera XT Index Kit, followed by an additional round of purification. Libraries were quantified using the Qubit 1x dsDNA HS Assay Kit (Invitrogen, USA) and pooled in equimolar concentrations. Sequencing was performed using the MiSeq v.3 (2 × 300 bp) chemistry kit. Adapter removal was carried out by the sequencer’s software. Generated raw 16S rRNA gene sequences were uploaded to the Sequence Read Archive (BioProject ID: PRJNA1240282).

### Bioinformatic analysis

The DADA2 pipeline in RStudio was used for quality filtering, error correction, and amplicon sequence variant (ASV) inference. Taxonomic classification was performed using the SILVA 138.1 ribosomal RNA database (silva_nr99_v138.1_wSpecies_train_set.fa). Relative abundances of bacterial genera were visualized using ggplot2 in RStudio, and statistical differences between swab types were analyzed using the Wilcoxon rank-sum test. Alpha diversity metrics (Shannon and Simpson indices) and beta diversity (Bray–Curtis dissimilarity) were computed using the phyloseq package in R. Principal Coordinates Analysis (PCoA) was performed to assess community composition differences. Statistical tests were performed using PERMANOVA in the adonis2 function of the R Vegan package (ver 2.6–4). Variation in sample microbial diversity in relation to sample type, year, and age class was visualized using a heatmap and cluster analysis implemented using the pheatmap package in RStudio 4.4.3 (2025-02-28 ucrt).

## Results

### Taxonomic composition of the affected swan microbiome

**Figure 1:**
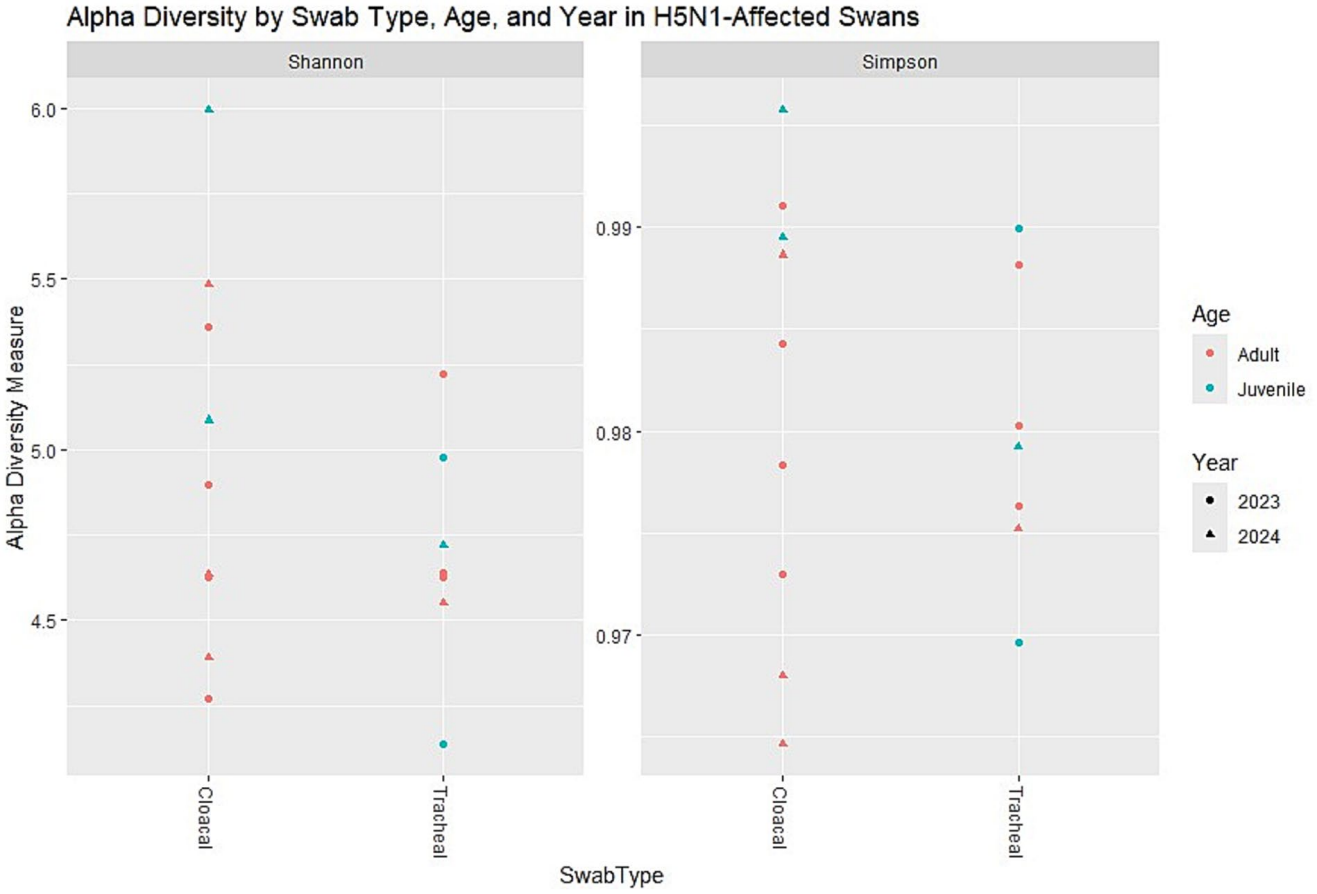
Relative abundance at the genus level. The most abundant genus appears to be one represented in brownish-green. Other noticeable genera include those in green, pink, and blue, but they are present in lower proportions. *n* = 16 samples (9 cloacal, 7 tracheal; 11 adults, 5 juveniles).

Analysis of taxonomic composition at the genus level revealed an obvious dominance of specific bacterial taxa across the dataset (Figure 1). Campylobacter emerged as the overwhelmingly predominant genus in both cloacal and tracheal samples, followed by Fusobacterium as the second most abundant taxon (Supplementary Table S1). Other consistently prevalent bacteria included Streptobacillus and Bacteroides, which are commonly associated with gut and mucosal microbiota. This pattern of dominance remained remarkably consistent in all sixteen examined samples, suggesting a systemic influence of these bacteria throughout the respiratory and digestive systems of the infected swans.

The visualization of relative abundances through stacked bar plots confirmed this taxonomic profile, with Campylobacter (represented in mustard yellow) constituting the majority of the bacterial community in all sample types, while other genera were present in substantially lower proportions. This consistent dominance across sample types represents a significant finding, as it suggests that H5N1 infection may facilitate similar patterns of microbial dysbiosis regardless of the physiological environment within the host.

### Microbial diversity patterns in H5N1-infected swans

The analysis of alpha diversity metrics revealed distinct patterns in the microbial communities of swans that were affected by H5N1 infection. Both Shannon and Simpson diversity indices consistently demonstrated that cloacal samples harbored more diverse microbial communities compared to tracheal samples across the dataset. The Shannon index, which accounts for both richness and evenness of taxa, showed elevated values in cloacal samples regardless of the swan’s age category. Similarly, Simpson diversity values approached 1 for most samples, indicating communities with relatively balanced compositions despite being dominated by a few key taxa.

**Figure 2:**
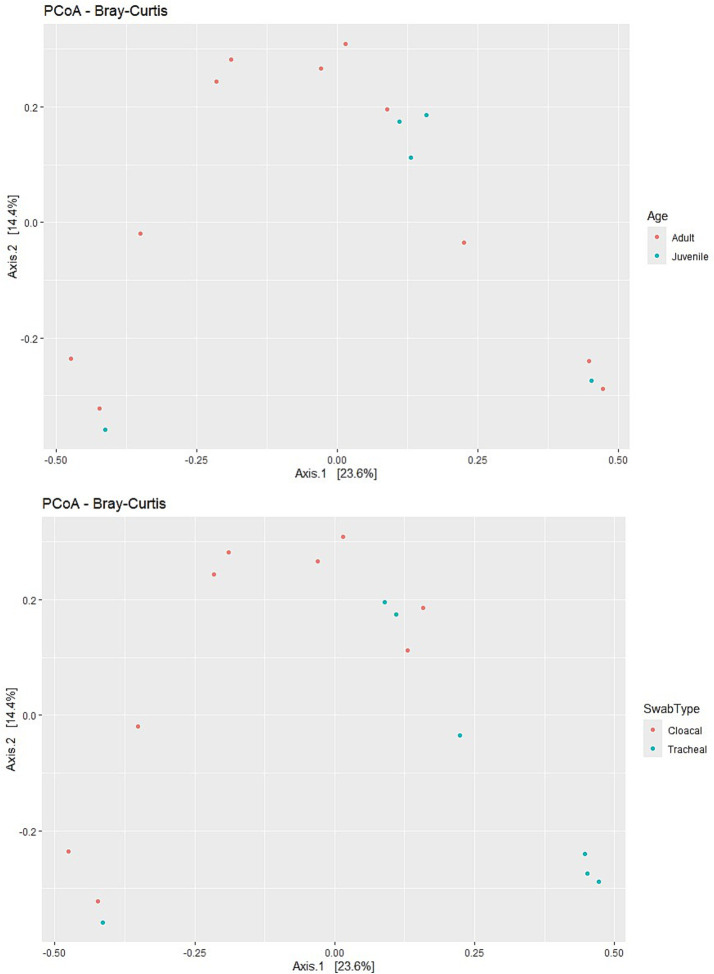
Alpha diversity metrics (Shannon and Simpson indices) across different factors: Swab type (cloacal vs. tracheal). Age (adult vs. juvenile). Year (2023 vs. 2024). 1. Shannon diversity (Left Panel)—cloacal samples tend to have slightly higher Shannon diversity than tracheal samples. Juvenile samples (blue) generally show a wider range of diversity compared to adults (red). Samples from 2024 (triangles) appear more variable than those from 2023 (dots). *n* = 16 samples (9 cloacal, 7 tracheal; 11 adults, 5 juveniles).

Age-related differences were apparent in the diversity metrics, with juvenile swans exhibiting a notably wider range of diversity values compared to adult birds. This variability suggests that younger swans potentially host more heterogeneous microbial communities, which could be attributed to differences in immune system development or varied environmental exposures prior to infection. Interestingly, when comparing samples collected in 2023 versus 2024, no pronounced year-based clustering was evident, indicating relatively stable microbial diversity patterns across the 2 years of the outbreak despite potential environmental or viral strain variations (Figure 2).

### Community composition and beta diversity analysis

**Figure 3:**
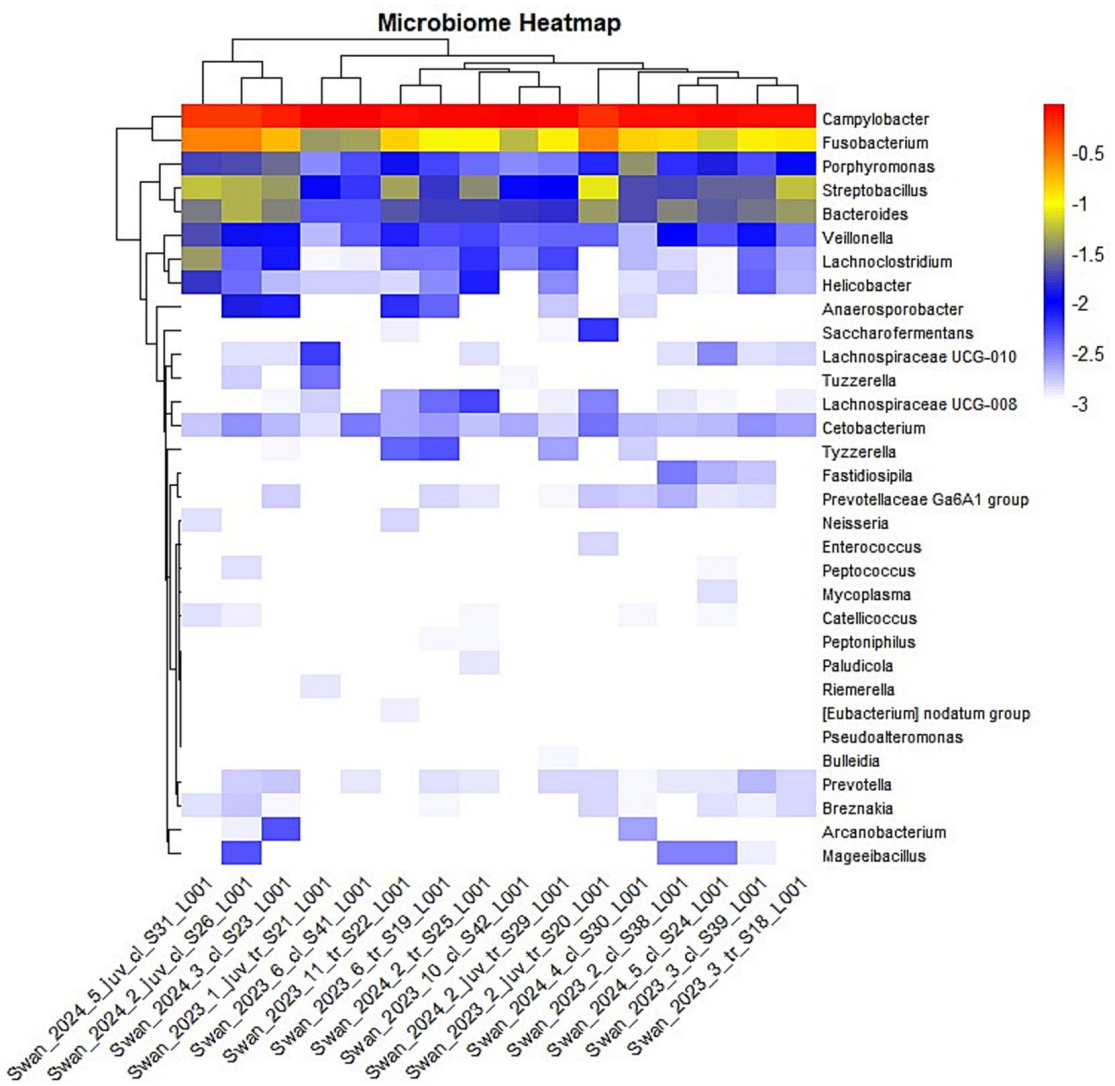
Analysis of alpha and beta diversity in H5N1-affected swans. Swab type effect—Cloacal swabs generally show slightly higher Shannon and Simpson diversity compared to tracheal swabs. Age effect—juveniles (blue) tend to have a broader range of diversity values compared to adults (red). Year effect—the shape differentiation suggests data is from multiple years (2023: circles, 2024: triangles). No strong year-based clustering is evident. *n* = 16 samples (9 cloacal, 7 tracheal; 11 adults, 5 juveniles).

Principal Coordinates Analysis (PCoA) based on the Bray–Curtis dissimilarity matrix revealed significant patterns in community structure across various sample categories. The visualization showed moderate separation between cloacal and tracheal samples, indicating that the sampling site is a major driver of microbial community differences in H5N1-infected swans. This anatomical differentiation was more pronounced than any clustering based on age categories, as juvenile and adult samples exhibited considerable overlap in the ordination space (Figure 3).

PERMANOVA testing provided statistical confirmation of these observed patterns. The analysis revealed that swab type was indeed a significant factor (*F* = 2.337, R^2^ = 0.143, *p* = 0.01), explaining approximately 14.3% of the variation in microbial composition across samples. Surprisingly, age did not significantly influence microbial composition (*F* = 0.6495, R^2^ = 0.044, *p* = 0.865), while year of collection showed only a marginal effect (*F* = 1.5349, R^2^ = 0.099, *p* = 0.098). Collectively, the tested factors (swab type, age, and year) explained approximately 29.67% of the total variation in microbial community structure (*F* = 1.6877, R^2^ = 0.29673, *p* = 0.008), indicating that additional unexplored factors likely contribute to the observed microbial diversity in these H5N1-affected birds.

### Heatmap visualization of microbial abundances

**Figure 4:**
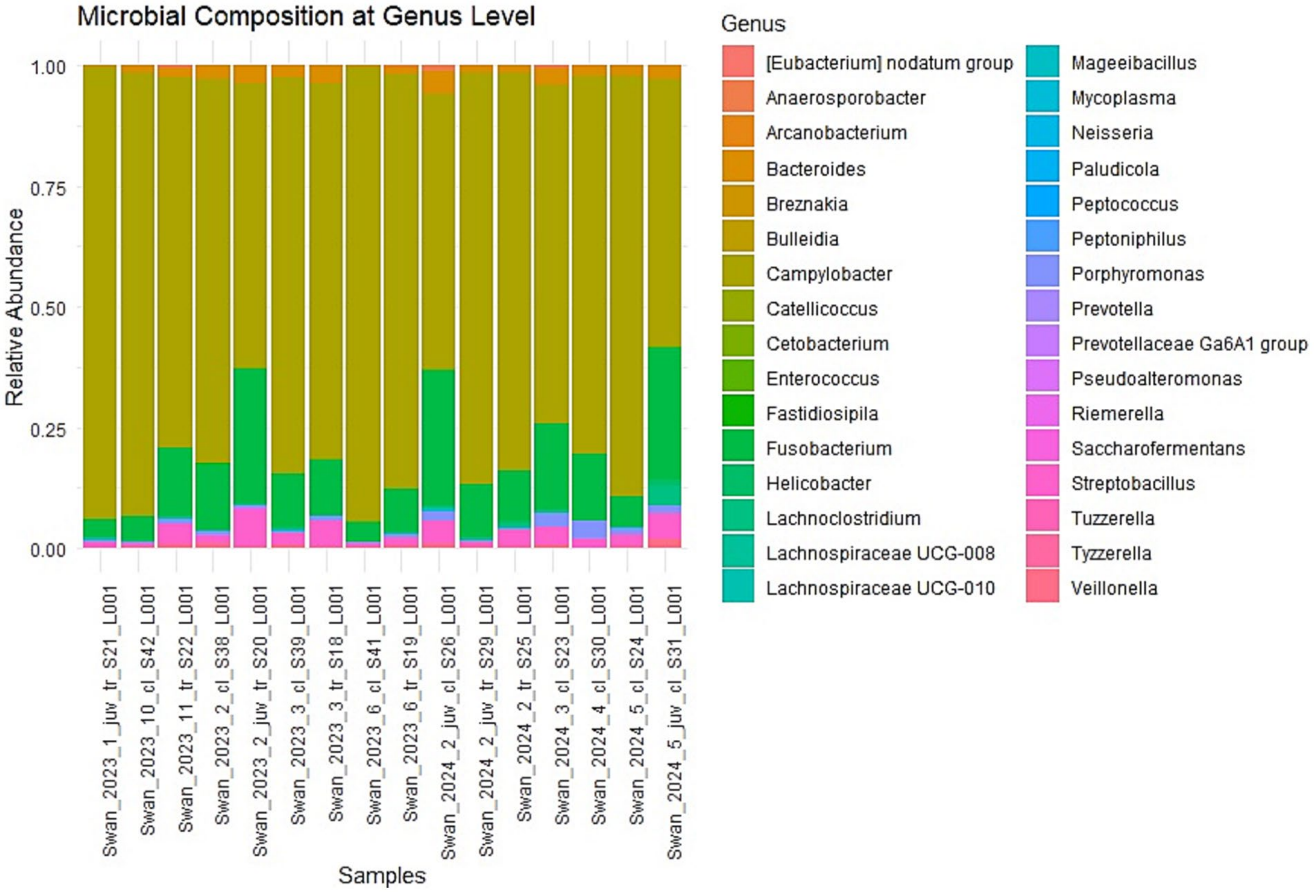
The heatmap visualization of the genus-level microbiome composition across multiple swan samples. Hierarchical clustering is applied to both rows (genera) and columns (samples), grouping similar patterns together. The log10 transformation of values helps normalize data and highlight differences in relative abundance. Color Interpretation—the color gradient represents the transformed abundance values: Red—highest abundance. Yellow—moderately high abundance, Blue—Low abundance, White—Very low or near-zero abundance. *n* = 16 samples (9 cloacal, 7 tracheal; 11 adults, 5 juveniles).

The hierarchical clustering analysis visualized through a heatmap further illustrated the patterns of microbial distribution across samples (Figure 4). The color gradient, ranging from red (highest abundance) through yellow (moderate abundance) to blue (low abundance), effectively demonstrated that again, Campylobacter and Fusobacterium genera maintained high abundance across most samples while others showed more variable or sample-specific patterns. Log10 transformation of abundance values normalized the data and highlighted differences in relative abundance across the dataset. The heatmap clustering suggested that while anatomical sampling sites (cloacal versus tracheal) influenced overall community structure, certain bacterial signatures were consistently associated with H5N1 infection regardless of other variables. This observation supports the hypothesis that avian influenza infection may drive predictable shifts in the avian microbiome, potentially contributing to disease pathogenesis. This heatmap successfully highlights microbiome composition differences across samples.

## Discussion

### Predominant bacterial genera in H5N1-infected swans

The most striking finding of this study was the overwhelming dominance of Campylobacter and, to a lesser extent, Fusobacterium in the microbiome of swans that died from H5N1 infection. Campylobacter and Fusobacterium emerged as the predominant genera in all examined samples, suggesting a systemic spread throughout the birds’ bodies. Their significant presence in all samples raises questions about their potential involvement in tissue damage following H5N1 infection. Supposedly, high abundances of Campylobacter and Fusobacterium in the swan samples are a consequence of dysbiosis rather than an increase in their pathogenicity. Campylobacter species are known to inhabit avian intestinal tracts, often as commensals, but typically represent a much smaller proportion of the normal gut microbiome in healthy birds (11). These bacteria are recognized as important zoonotic pathogens, with wild birds serving as natural reservoirs for species that can cause gastroenteritis in humans (12, 13). In poultry and waterfowl, Campylobacter commonly colonizes their guts without causing clinical signs. Exceptions such as C. hepaticus and C. bilis can cause “spotty liver disease” in layer hens, but these are specialized cases (14).

Fusobacterium, the second most abundant genus identified, comprises anaerobic bacteria that are normally minor components of avian gut microbiota (15) but in some microbiomes of wild bird species, it can be one of the common species (16). Thus, further research is needed to substantiate the significant increase of these bacteria in swans as a result of infection with the highly pathogenic H5N1 influenza virus.

Streptobacillus and Bacteroides were also consistently detected, though at lower abundances. Streptobacillus is less commonly reported in avian microbiome studies (16), while Bacteroides species are typical members of healthy gut communities in many vertebrates, including birds, where they play important roles in carbohydrate metabolism (17).

### Statistical confirmation of community patterns

The PERMANOVA analysis provided robust statistical confirmation of observed patterns in microbial community structure. Swab type emerged as a significant factor (*F* = 2.337, R^2^ = 0.143, *p* = 0.01), accounting for approximately 14.3% of the variation in microbial composition across samples. This finding indicates that despite the consistent dominance of certain genera across sample types, there remain significant anatomical differences in microbial communities between the respiratory and digestive systems of H5N1-infected swans. Interestingly, age did not significantly influence microbial composition (*F* = 0.6495, R^2^ = 0.044, *p* = 0.865), suggesting that H5N1 infection may override age-related differences in the microbiome that would typically be observed in healthy birds. The year of collection showed only a marginal effect (*F* = 1.5349, R^2^ = 0.099, *p* = 0.098), indicating relatively stable microbial patterns across the 2 years of the outbreak. Collectively, the tested factors explained approximately 29.67% of the total variation in microbial community structure, suggesting that additional factors not captured in this study, such as viral load, individual variation, or environmental conditions, also contribute to the observed patterns.

### Comparison with previous studies on H5N1-infected swan and other avian species microbiomes

Our findings differ significantly from those reported by Zhao et al. (10), who investigated the influence of H5N1 virus infection on the fecal microbiota of migrating whooper swans. While they observed shifts in microbial communities following H5N1 infection, they did not report the overwhelming dominance of Campylobacter that we identified. Instead, Zhao et al. found that H5N1 infection significantly increased the relative abundance of Aeromonas while decreasing Lactobacillus in the gut microbiota. The discrepancy between our results and those of Zhao et al. could reflect differences in sample collection timing (live birds versus freshly dead birds), geographical variables influencing baseline microbiome composition, or variations in viral strains and their effects on host–microbe interactions. Additionally, Zhao et al. focused exclusively on fecal samples, whereas our study incorporated both cloacal and tracheal samples, providing a more comprehensive view of microbial changes throughout the avian body following lethal H5N1 infection in swans.

Avian influenza virus compromises gut integrity, disturbs microbial balance, and triggers inflammation in the intestinal mucosa (18). In poultry, even low-pathogenic avian influenza virus (H9N2) leads to gut dysbiosis, characterized by reduced microbial alpha-diversity during the acute phase of infection and a delayed return to microbial equilibrium (8, 19). The severe illness observed in influenza-bacterial co-infections is primarily driven by impaired antibacterial immune responses and synergistic interactions between the pathogens. (20, 21).

### Comparison with normal avian microbiome

The microbiome composition observed in our H5N1-infected swans deviates substantially from the typical avian gut microbiome. Healthy bird gastrointestinal tracts are generally dominated by members of Firmicutes, Bacteroidetes, and Proteobacteria (22), with considerable variation across species, diet, and habitat. In contrast, our findings show an extreme dominance of specific genera (particularly Campylobacter and Fusobacterium) that are usually present in much lower abundances in healthy birds. This pattern suggests severe dysbiosis associated with H5N1 infection. The consistent presence of these potentially pathogenic bacteria across both respiratory and digestive tracts indicates a profound disruption of the normal microbiome throughout multiple body systems. The altered microbiome likely contributes to disease pathogenesis either through direct pathogenic effects or by compromising normal physiological functions dependent on healthy microbial communities, such as nutrient absorption, barrier protection, and immune regulation.

### Significance of findings

To our knowledge, this study represents the first comprehensive characterization of the microbiome in swans that died specifically from H5N1 infection. Our findings reveal a consistent pattern of microbial dysbiosis characterized by the dominance of Campylobacter and Fusobacterium genera across different anatomical sites. These results provide important insights into how highly pathogenic avian influenza may interact with the host microbiome, potentially contributing to disease severity and mortality. The identification of specific bacterial signatures associated with fatal H5N1 infection could potentially serve as markers for disease progression or indicators of poor prognosis in infected birds. Furthermore, our findings suggest that considering bacterial co-infections or secondary bacterial overgrowth may be important in understanding the full pathology of H5N1 infection in wild bird populations. The consistency of these microbial patterns across samples collected in different years (December 2023 and December 2024) indicates a stable host-pathogen-microbiome interaction that may be characteristic of H5N1 outbreaks in swan populations in the Caspian region.

### Limitations

A notable limitation of this study is that samples were collected from dead swans, which could potentially influence the microbiome composition due to post-mortem changes. However, several factors mitigate this concern. Samples were collected in cold December conditions (0–2°C), which would significantly slow bacterial overgrowth and post-mortem decomposition processes. Additionally, we specifically selected freshly dead swans, with some samples collected from birds that had died the same day or were in the process of dying. The carcass condition was assessed as “very fresh,” further minimizing potential post-mortem alterations. The immediate storage of samples in liquid nitrogen at −195°C would have effectively halted any further microbial changes after collection. While acknowledging this limitation, we believe the consistent patterns observed across multiple samples and the extreme dominance of specific taxa suggest genuine infection-associated dysbiosis rather than post-mortem artifacts. A notable limitation is the absence of a healthy control group, which prevents definitive conclusions that the observed microbiome changes are solely due to H5N1 infection and not influenced by other factors. Future studies including samples from healthy swans and birds at different stages of infection would further validate these findings.

## Conclusion

This study provides novel insights into the microbiome alterations associated with fatal H5N1 infection in wild swans in the Caspian Sea region. The consistent dominance of Campylobacter and Fusobacterium across different sampling sites suggests a profound and systemic impact of viral infection on the avian microbiome. These findings extend our understanding of how highly pathogenic avian influenza affects host–microbe interactions in wild birds, potentially identifying new factors that influence disease severity and mortality in these vulnerable populations. Further research into the specific mechanisms by which H5N1 infection drives these microbiome changes and how these alterations contribute to disease pathogenesis will be valuable for expanding our knowledge of avian influenza ecology and may inform future approaches to wildlife disease management.

## Data Availability

The original contributions presented in the study are publicly available. Generated raw 16S rRNA gene sequences were uploaded to the Sequence Read Archive (BioProject ID: PRJNA1240282).

## Supplementary material

SUPPLEMENTARY TABLE S1 Individual sample-level abundances in each swan sample.
